# Novel Selenium-based compounds with therapeutic potential for SOD1-linked amyotrophic lateral sclerosis

**DOI:** 10.1016/j.ebiom.2020.102980

**Published:** 2020-08-30

**Authors:** Kangsa Amporndanai, Michael Rogers, Seiji Watanabe, Koji Yamanaka, Paul M. O'Neill, S. Samar Hasnain

**Affiliations:** a Molecular Biophysics Group, Department of Biochemistry and System Biology, Institute of System, Molecular and Integrative Biology, Faculty of Health and Life Sciences, University of Liverpool, Liverpool, L69 7ZB, United Kingdom; b Department of Chemistry, Faculty of Science and Engineering, University of Liverpool, Liverpool, L69 7ZD, United Kingdom; c Department of Neuroscience & Pathobiology, Research Institute of Environmental Medicine, Nagoya University, Furo-cho, Chikusa-ku, Nagoya, 464-8601, Japan; d Department of Neuroscience and Pathobiology, Nagoya University Graduate School of Medicine, Aichi, 466-8550, Japan

**Keywords:** SOD1, Ebselen, Drug design, Neurodegeneration, Motor neuron disease, COVID-19

## Abstract

**Background:**

Amyotrophic lateral sclerosis (ALS), also known as motor neuron disease as well as Lou Gehrig's disease, is a progressive neurological disorder selectively affecting motor neurons with no currently known cure. Around 20% of the familial ALS cases arise from dominant mutations in the *sod1* gene encoding superoxide dismutase1 (SOD1) enzyme. Aggregation of mutant SOD1 in familial cases and of wild-type SOD1 in at least some sporadic ALS cases is one of the known causes of the disease. Riluzole, approved in 1995 and edaravone in 2017 remain the only drugs with limited therapeutic benefits.

**Methods:**

We have utilised the ebselen template to develop novel compounds that redeem stability of mutant SOD1 dimer and prevent aggregation. Binding modes of compounds have been visualised by crystallography. *In vitro* neuroprotection and toxicity of lead compounds have been performed in mouse neuronal cells and disease onset delay of ebselen has been demonstrated in transgenic ALS mice model.

**Finding:**

We have developed a number of ebselen-based compounds with improvements in A4V SOD1 stabilisation and *in vitro* therapeutic effects with significantly better potency than edaravone. Structure-activity relationship of hits has been guided by high resolution structures of ligand-bound A4V SOD1. We also show clear disease onset delay of ebselen in transgenic ALS mice model holding encouraging promise for potential therapeutic compounds.

**Interpretation:**

Our finding established the new generation of organo-selenium compounds with better *in vitro* neuroprotective activity than edaravone. The potential of this class of compounds may offer an alternative therapeutic agent for ALS treatment. The ability of these compounds to target cysteine 111 in SOD may have wider therapeutic applications targeting cysteines of enzymes involved in pathogenic and viral diseases including main protease of SARS-Cov-2 (COVID-19).

**Funding:**

Project funding was supported by the 10.13039/100000971ALS Association grant (WA1128) and Fostering Joint International Research (19KK0214) from the 10.13039/100009950Ministry of Education, Culture, Sports, Science and Technology (10.13039/501100001700MEXT), Japan.

**Research in Context**

**Evidence before this study**

Toxic inclusion of superoxide dismutase 1 (SOD1) mutant in neuron cells is one of the causes of Amyotrophic lateral sclerosis (ALS), a progressive neurological disorder. There are only two approved drugs, riluzole and edaravone, for ALS treatment with limited effectiveness. Discovery and developmentt of new drugs is urgently needed for ALS patients. Ebselen, a synthetic organo-selenium drug with potent anti-oxidant and cytoprotective effects, is considered to be a potential template for developing therapeutic molecules that can stabilise dimers of ALS-causing SOD1 mutant via binding to cysteine 111 which could reduce aggregation and delay disease progression.

**Added value of this study**

In this work, we established a group of organo-selenium compounds using benzoisoselenazolone warhead of ebselen with potent SOD1 mutant stabilisation effect. We elucidated binding modes of compounds at the dimer interface of SOD1 mutant by high resolution crystal structures which are beneficial for describing structure-activity relationship. Some of these compounds exhibited better *in vitro* neuroprotection in mouse neuronal cells than edaravone. *In vivo* disease onset delay by ebselen has been demonstrated for the first time in transgenic ALS mice model.

**Implications of all the available evidence**

Our findings suggest ebselen-based compounds are a valuable class of ALS therapeutic agents with superior *in vitro* neuroprotection than edaravone and with acceptable safety characteristics. *In vivo* disease onset delay of ebselen also strengthens the potential of ebselen-based compound to be progressed to next stage of drug development programme with potential for therapies that could be offered to patients as alternative treatments alone or in combination with other approved ALS drugs in future.

Alt-text: Unlabelled box

## 1 Introduction

Amyotrophic lateral sclerosis (ALS), also referred to as motor neuron disease (MND) and Lou Gehrig's disease, occurs in approximately 2 per 100,000 individuals worldwide, causing loss of motor neurons in the cerebral cortex and spinal cord resulting in severe muscle weakness, paralysis and fatal respiratory failure within 2–5 years from disease onset [1]. For some sporadic (sALS) and twenty percent of the familial (fALS) cases, the disease has its origins in superoxide dismutase whose stability as a dimer is compromised leading to a ‘gain of function’ that results in aggregation of protein [2]. Among the 25 genes contributing to fALS [1], genetic defect in *sod1* located on chromosome 21q22.11 was the first one to be discovered and is thus best studied with over 180 different known mutations [3], some of which are more common in certain populations. In North America, half of *sod1*-related fALS cases arise from mutation of Ala4 to Val [4], with one of the most severe forms of fALS with the shortest survival time [5]. To date, there are only two drugs approved for clinical use in ALS patients: riluzole and edaravone [6]. Riluzole modestly extends life expectancy by 2–3 months in the final stage of ALS but has poor positive effect at earlier stages of the disease [7]. Edaravone has had limited success and acceptance as a drug worldwide but is more effective than riluzole in slowing disease progression, with a limited application requiring daily intravenous infusion [8]. Hence, extensive research efforts continue globally in search for convenient and affordable new drugs that increase survival time with minimal adverse effect.

The copper- and zinc-containing superoxide dismutase 1 (SOD1) possesses four cysteine residues. The active and stable SOD1 is a homodimer stabilised by an intramolecular disulphide bond between cys57 and cys146 and metal sites bridged by histidine residues that are loaded with Cu and Zn [9]. SOD1 possesses two additional free cysteine residues cys6 and cys111, is mostly distributed in cytoplasm and is responsible for converting toxic reactive superoxide into oxygen and hydrogen peroxide preventing oxidative stress within the cells [10]. Extensive efforts have established that it is not the functional loss of SOD's enzymatic activity that is responsible for the ALS causing properties [11], but destabilisation of dimer caused by mutations or loss of metals in the metal site that leads to aggregation properties which are the disease causing agents [9,12]. Moreover, ALS-causing mutants including A4V have been found to heterodimerise with wild-type SOD1 and exert higher accumulation of toxic SOD1 aggregates in cell and animal models [13,14]. Several small molecules have shown stabilising effect on SOD1 dimer and diminished inclusions which could delay the development of ALS [15], [16], [17]. One of the binding sites within SOD1 structure focused as a drug target is cys111, which is a solvent-exposed residue at the dimer interface that is vulnerable to oxidative modification giving rise to aggregation [18,19]. Chemical cross-linking between subunits at cys111 using maleimide-based compound has been proven to be a good strategy to promote thermal stability of G93A and G85R SOD1 [15]. Repurposing chemotherapeutic agent of cisplatin for curing neurodegenerative disease also raises SOD1 dimer stability and inhibits oligomerisation of metal-free SOD1 [16]. However, both compounds could not be developed further since the steric hindrance imposed by drug protein adduct formation negatively influences the interaction of SOD1 with human copper chaperone (hCCS) resulting in the disruption of SOD1 maturation with resultant toxicity [20].

Ebselen is an organo-selenium compound that scavenges reactive oxygen species and enhances cellular defence from oxidative damage [21]. Neuroprotective actions of ebselen in rodent models have been observed in several reports [22], [23], [24]. Notably in proteomics studies, ebselen can compromise the cellular toxic effect in mitochondria induced by the expression of ALS-related SOD1 mutant [25]. Furthermore, ebselen exhibits covalent binding at cys111 without impeding interaction between SOD1 and hCCS indicating greater potential over other compounds to be developed as an effective neuroprotective drug [17].

In our recent work, differential scanning fluorescence (DSF) assay has been introduced as a medium-throughput screen for ebselen and some potential derivatives that promote SOD1 thermal stability [26]. Here, we have developed next generation ebselen derivatives based on the compounds previously published with improved SOD1 dimer stabilising effect and drug-like properties. A new higher diffracting form of small molecule-bound A4V SOD1 crystals has been established allowing us to visualise ligand pose of the compounds targeting cys111 in the pathogenic target enabling lead optimisation through structure-based approaches. Furthermore, ebselen and our potent derivatives were investigated for neuroprotective effects in *in vitro* and *in vivo* models. Some compounds at nanomolar concentration have been demonstrated to redeem *in vitro* viability of mouse neuron cells expressing pathogenic SOD1 including A4V mutant up to similar level of the wild-type SOD1 counterpart. We show that ebselen clearly replicates neuroprotective activity *in vivo* by delaying disease onset in G93A SOD1 transgenic mice, which is the representative mouse model for SOD1-linked ALS [27].

## 2 Materials and methods

### 2.1 Ethics statement

The experiments using genetically modified animals and organisms were approved by the Animal Care and Use Committee and the recombinant DNA experiment committee of Nagoya University (approval numbers #19269 and #143, respectively). The mice were treated in compliance with ARRIVE guideline and the animal use guidelines of the Animal Care and Use Committee, Nagoya University.

### 2.2 Synthesis of lead compounds

Details for the synthesis of ebselen analogues are included in the supplementary information.

### 2.3 Recombinant SOD1 production

The pET303C plasmids containing A4V and double-point mutated A4V C6S human SOD1 genes were generated by site-directed mutagenesis from wild-type gene using primers shown in Table S1 and transformed into *E. coli* strain BL21(DE3). The SOD1 transformants were cultured at 37 °C in ampicillin-supplemented LB broth until optical density at 600 nm reaches 0.6–0.8. Then, SOD1 expression was induced by the addition of 0.5mM Isopropyl β-d-1-thiogalactopyranoside (IPTG) and 0.3mM ZnSO_4_ followed by incubation at 18°C for 16 hours. Recombinant proteins were purified by two different methods previously described [28]. A4V^C6S^ SOD1 mutant used in DSF assays was purified using anion-exchange chromatography on diethylaminoethanol (DEAE) sepharose and eluted in a stepwise increasing concentration of NaCl. Protein fractions eluted in 5–100mM NaCl were combined and loaded to a Superdex 200 16/600 column (GE Healthcare) in Tris-buffered saline (TBS) pH 7.4. Recombinant A4V SOD1 used in crystallographic study was purified by precipitation cut using 2.5M ammonium sulphate followed by hydrophobic interaction chromatography on Phenyl sepharose (GE Healthcare) eluted in a stepwise decreasing concentration of ammonium sulphate. A4V SOD1 obtained in 1-2M ammonium sulphate fractions were dialysed against Tris-buffered saline (TBS) pH 7.4 at 4 °C overnight followed by gel filtration on a Superdex 200 16/600 column (GE Healthcare) eluted with the same buffer. The purified proteins were snap-frozen in liquid nitrogen and stored at −80 °C prior to experiments. The concentration of all SOD1 proteins was determined by ultraviolet absorption at 280nm using a molar extinction of 5500M^−1^cm^−1^.

### 2.4 Differential scanning fluorimetry (DSF)

All DSF experiments were carried out using a StepOnePlus Real-Time PCR machine (Life Technologies). 100 μM A4V^C6S^ SOD1 was pre-incubated with 500 μM compounds diluted from 250mM stock solution at 4°C overnight. The mixtures were buffer-exchanged against fresh TBS using micro-centrifugal ultrafilter (Vivaspin, MWCO 5kDa) to remove excess compound. 70 μM buffer-exchanged protein was mixed with 10 × concentration of SYPRO Orange dye (Life Technologies) which was used as a fluorescent probe. Fluorescent intensities were monitored from 25 °C to 95 °C at a ramp rate of 0.3 °C/min. The melting temperature (T_m_) was calculated based on the Boltzmann equation using the T_m_ Tool^TM^ software (Life Technologies)

### 2.5 Crystallisation and structure determination

All compounds were prepared for 250mM stock solution by dissolving in DMSO. 0.2mM of A4V SOD1 was incubated with 1mM compound being at 4 °C overnight before concentrated to 10mg/mL SOD1. 3 μL of A4V SOD1 solution was set hanging crystallisation drops by mixing with 3 μL of reservoir solution containing 100mM sodium acetate pH 4.7, 150mM NaCl and 2.5–2.8M ammonium sulphate. The crystallisation drops were incubated at 19°C for 2-3 days allowing vapour diffusion against corresponding reservoir solutions before macro-seeding using a few A4V SOD1 crystals in P2_1_ space group previously grown in the same condition. Mature crystals were obtained within a week after seeding and soaked in Paratone oil (Hampton Research) as cryo-protectant before freezing in liquid nitrogen. Diffraction data was collected at 100K on I03 (compounds **3, 6** and **9**), I04 (compounds **1** and **5**), I04-1 (ebselen, compounds **2** and **10**) and I24 (compound **13**) at Diamond Light Source, UK, integrated using DIALS [29] and scaled using Aimless [30] in CCP4 suite. Phase problem of all structures was solved by molecular replacement using an A4V SOD1 structure (PDB: 1UXM; chain A and B) as a starting model in MolRep [31]. Structure models were initially refined by rigid body refinement in Refmac5 [32] and then manually modified in COOT [33] followed by cycles of restrained refinement in Refmac5. Ligand models of ebselen-based compounds were produced in Jligand [34] and manually added into corresponding electron density.

### 2.6 Cell culture, transfection and MTS assay

Mouse neuroblastoma Neuro2a cells (RRID: CVCL_0470) were cultured and measured their viability as described previously [35]. Briefly, the cells seeded at 5.0 × 10^3^ /well on a poly-D-lysine coated 96 well plate were transfected to express mutant human SOD1 species using Lipofectamine 2000 (Thermo Fisher Scientific). After 6 h of transfection, the growth medium (Dulbecco's Modified Eagles' Medium (DMEM) containing 4.0 g/L glucose, 10 %(v/v) fetal bovine serum (FBS) (both from Thermo Fisher Scientific)) was replaced with a differentiation medium (DMEM containing 1.0 g/L glucose, 2 %(v/v) FBS and 2 mM N6,2′-O-dibutyryladenosine 3′,5′-cyclic monophosphate (Nacalai Tesque, Kyoto, Japan)) with the indicated ebselen-derived compounds at the indicated concentration. All the compounds were first dissolved into DMSO at 10 mM and stored at -30 °C until use. The cells were incubated for 48 h, and the differentiation medium was replaced with a fresh one. Then, the cell viability was measured using MTS assay (CellTiter 96® AQueous One Solution Cell Proliferation Assay, #G3580, Promega Biosciences, San Louis Obispo, CA, USA) according to the manufacturer's instructions with an absorption spectrophotometer Infinite F50R (TECAN Group Ltd., Männedorf, Switzerland). To determine LC_50_ of the compounds, the cell viability at different compound concentrations was fitted using the least squares method to a sigmoid curve with the following formula; $\left[Cell\;Viability\right]=\;\frac{100}{\left(1+10^{n(log9[LC50])-log(\left[Concentration\right])}\right)}$

### 2.7 Animals

Transgenic mice expressing SOD1^G93A^ (B6.Cg‐Tg(SOD1*G93A)1Gur/J) (RRID: IMSR_JAX:004435) were obtained from the Jackson Laboratory (Bar Harbor, ME, USA). Genotyping for the SOD1^G93A^ mice and determination of the times for disease onset and end-stage were previously described [36]. In brief, the disease onset was determined by the time when the mice reached maximal body weight. The mice were randomly divided into two groups that fed with a powdered CE-2 diet (CLEA Japan Inc., Tokyo, Japan) in the presence or absence of 0.016 % ebselen (#E0946, Tokyo Chemical Industry Co., Ltd., Tokyo, Japan) using a feeder for powder diet provided by Shinano Seisakusho Inc. (#SN-950) (Tokyo, Japan) from 70 days of age to the end-stage. The diet was replenished three times per week, and the mice had free access to their assigned diet and deionized water. We estimated the amount of ebselen taken by mice from the food intake, and confirmed that about 300 µg/kg/day ebselen was taken during all over the cohort. The mice were housed in the specific pathogen-free (SPF) environment (with a 12 h light‐dark cycle at 23 ± 1°C) and treated in compliance with the requirements of the Animal Care and Use Committee, Nagoya University.

### 2.8 Statistics

For animal experiments, G93A SOD1 mice were grouped in a random manner. Measurements of motor function and body weights were carried out in a non-blinded manner. Survival times were analyzed with a log-rank test, and no sample size calculation was performed in this study. All the data of MTS assays were analyzed by one-way ANOVA followed by the post-hoc Tukey's multiple comparison t-test. All the statistical analyses were carried out by using GraphPad Prism 8 software (GraphPad Software, La Jolla, CA).

### 2.9 Role of funding source

Compound synthesis, DSF screen and crystallography were supported by the ALS Association grant (WA1128). Cell-based and animal model studies were granted by Fostering Joint International Research (19KK0214) from the Ministry of Education, Culture, Sports, Science and Technology (MEXT), Japan. Funders did not play any part in study design, data collection, data analyses, interpretation, or writing of the manuscript.

## 3 Results

### 3.1 Generation of novel ebselen analogues with good physicochemical properties that promote SOD1 thermal stability

Ebselen has been demonstrated to bind cys111 and stabilise dimer of A4V SOD1, which is severely prone to monomerise and aggregate leading to acute form of ALS [17,26]. Based on benzoisoselenazolone core of ebselen, functionalised *N*-aryl and methylene linker moieties were incorporated into the organoselenium core to improve dimer stabilisation of SOD1 and drug-like properties (Figure S1). To rank stabilising effect of ebselen-based small molecules, DSF screen against A4V SOD1 with C6S mutation background (A4V^C6S^ SOD1) has been used to evaluate performance *via* different melting temperatures of compound-treated and native protein (∆T_m_). C6S background was designed to eliminate the destabilisation of A4V SOD1 in solution due to occasional binding of ebselen to free thiol of cys6 which results in undeterminable melting curve of DSF assay [26]. A greater positive ∆T_m_ indicates improved thermal stability of A4V^C6S^ SOD1 affected by compound binding. The compounds with a methylene linker imparted greater thermal stability than *N*-aryl derivatives. The structures of A4V SOD1 bound with ebselen and its methylene linked derivative (compound **2**) derived from C2 space group crystals reveal distinct binding poses of ligands (Figure S2), which could describe different outcomes in ∆T_m_ values. Ebselen, an *N*-aryl compound, interacts with loop VI of SOD1 (residues 101-109), whilst compound **2** poses perpendicularly to ebselen placing its aromatic tail into the gap between Thr2 and Ile151. Therefore, functionalising the aryl ring of the methylene linker scaffold that enables further interactions may provide better stabilisation of SOD1 dimer. This offers greater substrate scope to be incorporated into the methylene linker series compared to the *N*-aryl scaffold analogues [26].

Herein, a series of methylene linker (compounds **1–10** and **14**) and *N*-aryl (compounds **11–13**) compounds incorporating a variety of functional groups were synthesised and their ∆T_m_ values in A4V^C6S^ SOD1 were measured by DSF assays. Compounds **1,3** and **9** have stronger stabilising effect on A4V^C6S^ SOD1 above other methylene linked compounds as shown in Table 1. Furthermore, incorporation of a carbonyl morpholine moiety with benzoisoselenazolone core (compounds **12–14**) seems to establish outstanding ∆T_m_ values at the same level or better than ebselen. Apart from increasing stability of SOD1 dimer, potent candidates need to have suitable drug-like properties that allow central nervous system (CNS) access and desirable absorption, distribution, metabolism and excretion (ADME) profiles. These parameters can be predicted and ranked by multi-parameter optimisation (MPO) score using an algorithm developed by Pfizer [37]. The following physicochemical aspects are accounted to address specific ADME properties to ensure the compound is CNS penetrable based on known CNS drugs and potential candidates along with a diverse range of Pfizer compounds. MPO scores range from 0 to 6.0, and the values ≥4.0 represent likelihood of compounds to reach CNS drug target. Figure S3 shows the map of desirable ranges for each physicochemical parameter of the MPO scoring algorithm. Each parameter is given defined limits and if any parameter is found outside the defined limits then a penalty is given to each specific parameter which dictates the overall MPO score. All MPO scores and other calculated physicochemical parameters of each compounds are given in Tables 1 and 2 and highlighted how good or bad in traffic light system. It can be clearly seen that the compounds with high ∆T_m_ values, particularly compounds **3, 6, 9–14**, possess desirable ranges of MPO scores and other parameters, qualifying them for tests in cellular and animal models.

**Table 1:** Methylene linker series compounds **1**-**10** with their A4V^C6S^ SOD1 dimer stability (ΔT_m_), MPO score and each physicochemical parameter that contributes to the overall score. **1-3** are from [26]. A traffic light system was employed to demonstrate each value contribution; Green – good/desirable; Amber – ok; Red – poor/undesirable. ClogP: calculated logarithm of partition coefficient of a compound between n-octanol and water, ClogD: calculated logarithm of distribution coefficient of a compound between n-octanol and water at pH 7.4, MW: molecular weight, tPSA: topological polar surface area, CNS MPO: central nervous system multiparameter optimisation.

**Table 2:** *N*-aryl series compounds **11**-**14** with their A4V^C6S^ SOD1 dimer stability (ΔT_m_), MPO score and each physicochemical parameter that contributes to the overall score. A traffic light system was employed to demonstrate each value contribution; Green – good/desirable; Amber – ok; Red – poor/undesirable. ClogP: calculated logarithm of partition coefficient of a compound between n-octanol and water, ClogD: calculated logarithm of distribution coefficient of a compound between n-octanol and water at pH 7.4, MW: molecular weight, tPSA: topological polar surface area, CNS MPO: central nervous system multi-parameter optimisation.

### 3.2 Direct visualisation of binding modes of compounds in A4V SOD1

**Fig. 1:**
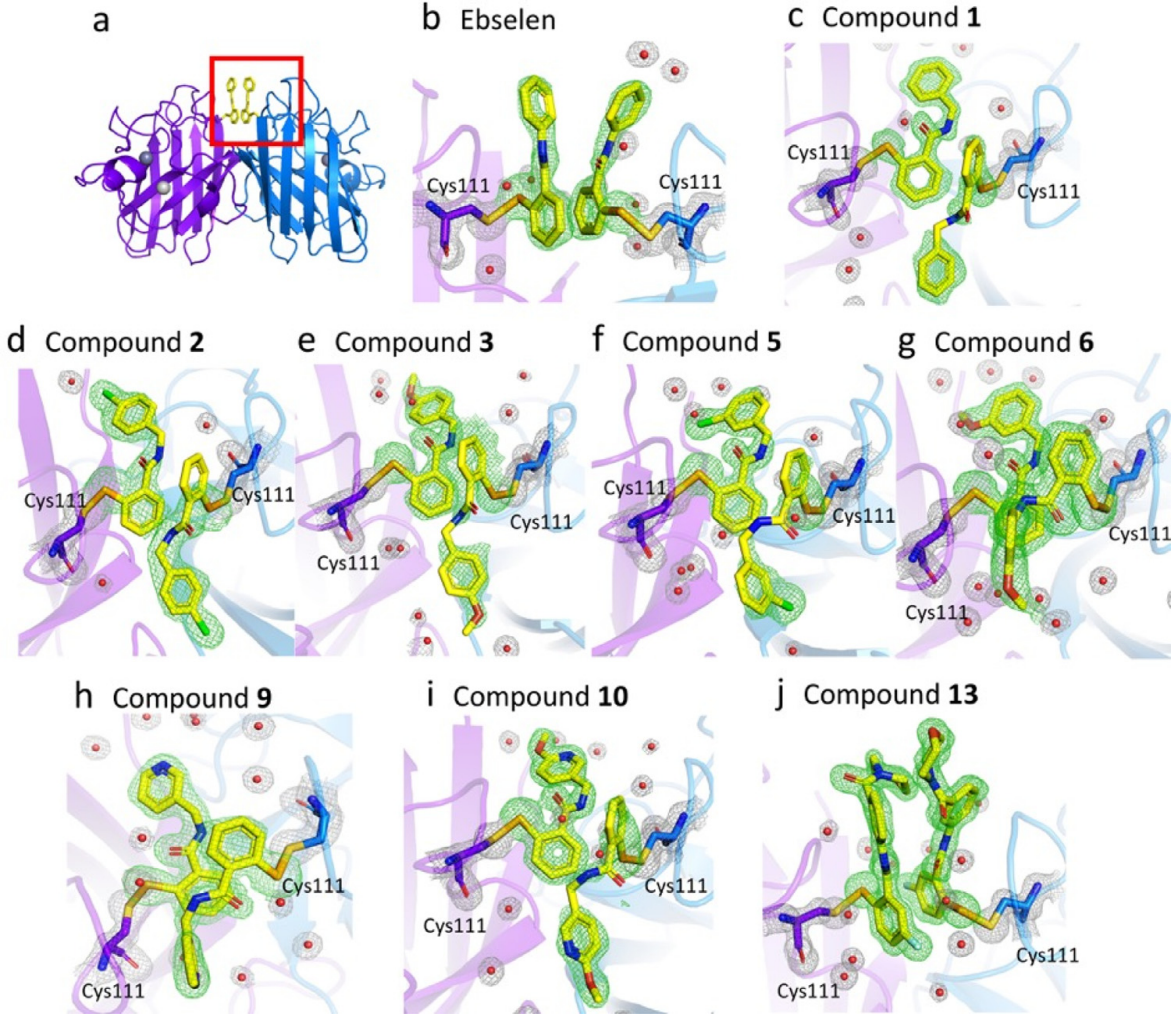
The crystal structures of A4V SOD1 in P2_1_ space group with bound compounds. **a** Cartoon representation of A4V SOD1 dimer with ligands. The ligands (yellow sticks in red box) bind each monomer (purple and blue) at cys111, located at the dimer interface. Electron density (2F_o_-F_c_) maps of **b** ebselen and **c-j** compounds **1, 2, 3, 5, 6, 9, 10** and **13** are contoured at 1σ in green mesh. The 2F_o_-F_c_ map of water molecules (red spheres) and cys111 residues (purple and blue sticks) are contoured at 1σ in grey mesh. (For interpretation of the references to colour in this figure legend, the reader is referred to the web version of this article.)

A4V SOD1 crystals in the recently obtained high resolution form in the P2_1_ space group enabled determination of several ligand-bound A4V SOD1 structures to a resolution range of 1.30-1.95Å depending on the compound and showing details of binding mode of these compounds at cys111. Crystallographic data collection and refinement statistics of P2_1_ crystals of A4V SOD1 with ebselen and eight derivatives are presented in Table 3. There is only one SOD1 dimer in asymmetric unit of P2_1_ crystals. Electron density maps of the ligands in this crystal form are clearly visible between dimer interface and able to be modelled unambiguously as shown in Fig. 1. With higher resolution diffraction of P2_1_ crystals, we can observe electron density of water molecules around the ligand, which is an advantage for investigating water bridge formation between ligand and amino acids that could rationalise how some compounds promote ∆T_m_ of SOD1. All co-crystallised compounds in P2_1_ space group also show that the direction of aromatic tail of each compound depends on its scaffold. Ebselen which is a *N*-aryl compound appears to pose their aromatic tail jutting out to the solvent space along Loop VI of SOD1, while compound **1** which contains methylene linker is placed perpendicularly to Loop VI and contacted with *N*- and *C*-termini at Thr2 and Ile151, respectively (Fig. S4). For other compounds in each scaffold, the overall ligand poses with the same scaffold are mostly similar to either ebselen or compound **1**.

**Table 3:** Crystallographic data collection and refinement statistics of A4V SOD1 with bound compound crystals in P2_1_ space group.

Parameter/ligand	Ebselen	Compound 1	Compound 2	Compound 3	Compound 5
Data collection					
Space group	P2_1_	P2_1_	P2_1_	P2_1_	P2_1_
Cell dimensions					
a, b, c (Å)	38.95, 67.72, 50.65	38.34, 67.95, 50.46	38.61, 68.29, 50.90	37.33, 68.26, 50.71	38.50, 68.32, 51.32
α, β, γ (°)	90.00, 106.40, 90.00	90.00, 105.54, 90.00	90.00, 105.76, 90.00	90.00, 105.07, 90.00	90.00, 106.36, 90.00
Resolution (Å)*	39.48–1.45 (1.47–1.45)	48.61–1.35 (1.37–1.35)	39.80–1.95 (2.00–1.95)	32.46–1.60 (1.63–1.60)	36.94–1.55 (1.58–1.55)
R_merge_*	4.6 (58.5)	4.8 (40.1)	6.5 (34.4)	7.1 (39.8)	4.2 (27.9)
I/σI*	10.6 (1.8)	9.7 (1.8)	7.4 (2.3)	6.9 (2.0)	11.6 (2.0)
CC1/2 (%)*	0.998 (0.686)	0.997 (0.795)	0.985 (0.538)	0.991 (0.774)	0.998 (0.887)
Completeness (%)*	99.3 (99.8)	99.8 (99.4)	99.7 (98.9)	100.0 (99.8)	99.8 (99.6)
Redundancy*	3.3 (3.4)	3.2 (2.6)	3.1 (3.2)	3.4 (3.4)	3.2 (3.3)
Refinement					
No. reflections	44,730	54,619	18,610	33,188	37,031
Rwork/Rfree	15.28/18.82	20.79/23.21	22.10/24.20	18.58/21.71	18.91/20.77
No. atoms					
Protein	2237	2291	2200	2289	2291
Ligand/ion Water	32/34 195	34/19 229	36/14 95	57/22 223	36/6 166
B-factors					
Protein	22.06	20.27	31.69	26.15	22.24
Ligand/ion	43.20/ 49.69	43.12/ 36.12	62.32/ 55.43	29.95/ 31.28	38.60/ 23.81
Water	31.28	31.43	36.22	28.55	29.98
R.M.S. dev.					
Bond lengths (Å)	0.0181	0.0151	0.0117	0.0118	0.0131
Bond angles (°)	1.912	1.8737	1.608	1.643	1.746
PDB code	6Z4G	6Z4O	6Z4H	6Z4I	6Z4J


Parameter/ligand	Compound 6	Compound 9	Compound 10	Compound 13	
Data collection					
Space group	P2_1_	P2_1_	P2_1_	P2_1_	
Cell dimensions					
a, b, c (Å)	38.24, 68.32, 50.98	38.93, 67.49, 50.78	38.41, 68.00, 50.90	39.07, 68.09, 50.86	
α, β, γ (°)	90.00, 106.17, 90.00	90.00, 106.12, 90.00	90.00, 106.24, 90.00	90.00, 106.87, 90.00	
Resolution (Å)*	34.33–1.45 (1.47–1.45)	34.69–1.50 (1.53–1.50)	36.88–1.55 (1.58–1.55)	39.60–1.25 (1.27–1.25)	
R_merge_*	3.1 (56.1)	5.8 (46.5)	6.5 (42.2)	7.8 (56.2)	
I/σI*	13.3 (1.4)	9.7 (1.9)	8.5 (2.0)	8.0 (2.2)	
CC1/2 (%)*	0.999 (0.609)	0.994 (0.745)	0.992 (0.798)	0.988 (0.524)	
Completeness (%)*	99.4 (99.4)	95.2 (82.0)	99.7 (99.7)	99.3 (99.3)	
Redundancy*	3.3 (3.0)	3.5 (3.1)	3.1 (3.1)	3.3 (3.2)	
Refinement					
No. reflections	44,648	40,405	36,517	66,385	
Rwork/Rfree	19.47/20.92	18.66/20.62	19.49/20.78	17.76/19.12	
No. atoms					
Protein	2244	2261	2286	2260	
Ligand/ion Water	38/6 232	34/19 232	38/11 190	50/29 288	
B-factors					
Protein	21.59	19.68	21.86	13.18	
Ligand/ion	29.89/ 23.64	29.57/ 28.64	36.52/ 34.53	22.21/ 26.92	
Water	29.62	28.13	31.30	24.02	
R.M.S. dev.					
Bond lengths (Å)	0.0147	0.0115	0.0103	0.0155	
Bond angles (°)	1.747	1.749	1.682	1.901	
PDB Code	6Z4K	6Z4L	6Z4M	6Z3V	

⁎ Values in parenthesis denote the highest resolution shell.

**Fig. 2:**
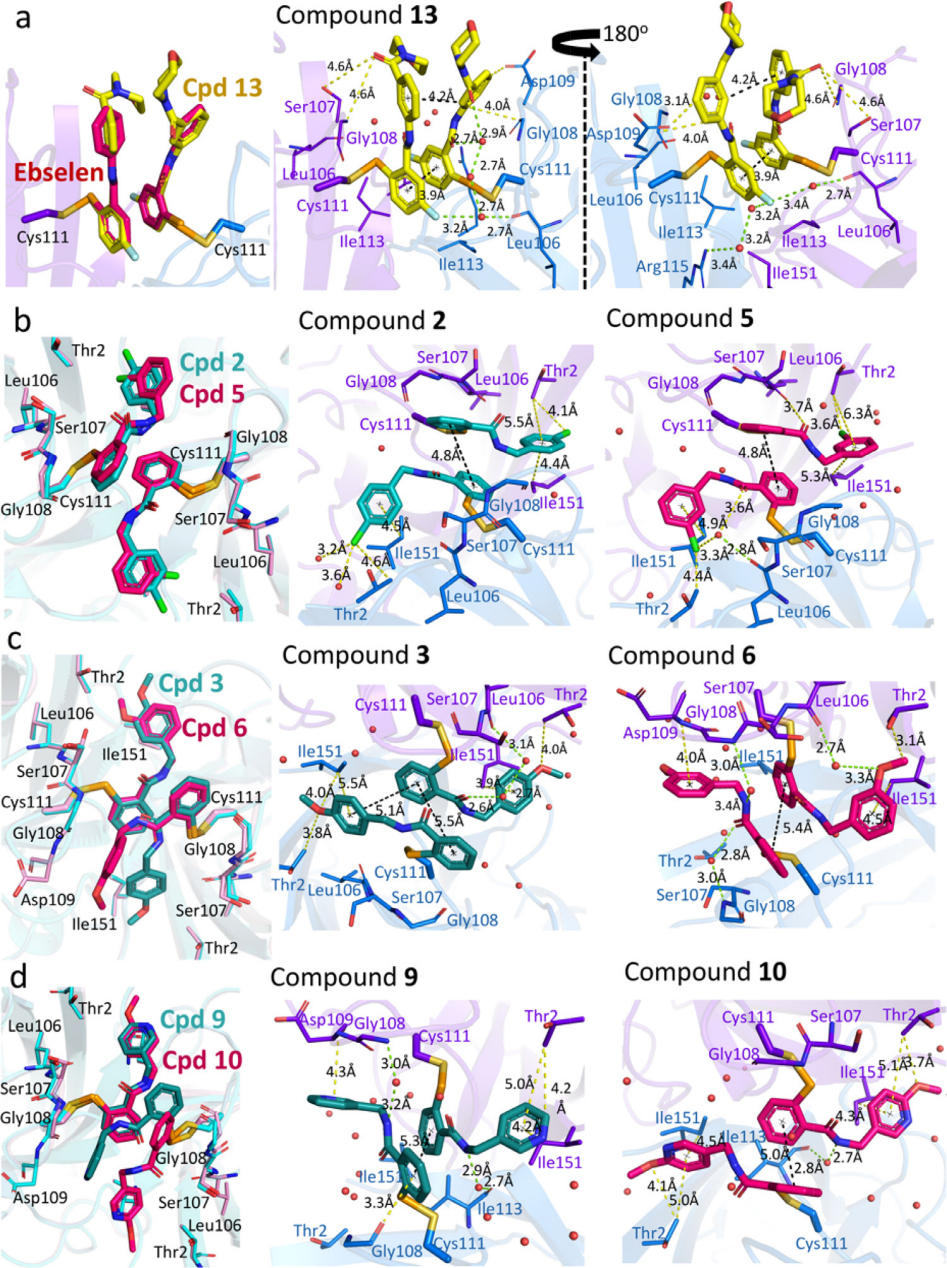
Superimposed and individual crystal structures of A4V SOD1 bound with compounds. **a** Ebselen (pink) and compound **13** (yellow). **b** Compounds **2** (cyan) and **5** (pink). **c** Compounds **3** (cyan) and **6** (pink). **d** Compounds **9** (cyan) and **10** (pink). Surrounding amino acid residues of each monomers are coloured in purple and blue. Waters are represented as red dots. Hydrogen bonds are illustrated as green dashed lines. The distances between atoms and between aromatic rings are illustrated as yellow and black dashed lines, respectively. (For interpretation of the references to colour in this figure legend, the reader is referred to the web version of this article.)

In crystal structures of A4V SOD1–ebselen, –*N*-aryl compound **13** (Fig. 2a), we clearly observe π-π stacking between aromatic rings of these compounds. The overlaid structures in Fig. 2a show that fluorinated seleno-head group of the compound **13** has an identical pose to ebselen, so incorporation with fluorine atom does not change the binding pose. Notably, the carbonyl morpholine moiety and fluorine atom in compound **13** can establish few hydrogen bonds with water molecules bridging between protein and ligand (Figs. 1a). These interactions explain the rationale behind greater ∆T_m_ of compound **13** above ebselen and could be utilised as the template for future lead optimisation. For the compounds with a methylene linker, we have successfully co-crystallised six of this class of compounds giving good opportunity to see the influence of Cl and OMe substituents on the phenyl or pyridyl tail group. Starting with chlorobenzyl ebselens, compounds **2** and **5** have a highly similar ligand pose observed in overlaid crystal structures in Fig. 2b. Unlike *N*-aryl compounds, A4V SOD1-compounds **2** and **5** structures display longer distance between seleno-aromatic rings (Fig. 2b). Thus, the interaction between compounds is weaker than *N*-aryl compounds. Moreover, *meta*-Cl substituent shifts the benzyl group away from Thr2 and Ile151 impairing hydrophobic contacts between *N*- and *C*- termini (Fig. 2b). This may cause weaker SOD1 stability of compound **5** than compound **2**. In the superimposed structures of A4V SOD1-compounds **3** and **6**, only one monomer appears perfectly overlaid compound molecules like compounds **2** and **5** (Fig. 2c). Compound **6** at another monomer acts as a bridge between monomers by localisation of its *meta*-methoxybenzyl tail closer to the main chain of Asp109 of another monomer, while compound **3** behaves identically to compounds **1, 2** and **5** whose tail picks up hydrophobic interactions with Thr2 and Ile151. Cross-monomer interaction of compound **6** seems to enhance dimer interface whereas ∆T_m_ of compound **6** is lower than compounds **1** and **3**, which have closer contact to *N*- and *C*-termini (Fig. 2c). This indicates that the interaction between Thr2 and Ile151 is probably more important for dimer stability than the interaction between benzyl tail and neighbour Asp109. On the other hand, this concept is not applicable to compounds **9** and **10** that pyridyl or *para*-methoxypyridyl rings were replaced in the scaffold of compound **1**. Strong stabilising profile was observed in both compounds, especially compound **9** with greater ΔT_m_ than ebselen. The binding pose of compound **9** appears in the same way as compound **6** but compound **10** performs similarly to compound **3** (Fig. 2d). This contrasting effect may occur because higher polarity of the pyridyl ring that can establish stronger cross-monomer linking than compound **6** *via* dipole-diploe interaction with the main chain of neighbour Asp119 (Fig. 2d) resulting in the best stabilisation among methylene linker series. Based on an integrated knowledge of the binding mode visualisation in crystallographic structures, DSF assays and MPO score analysis, we noted that compounds **9** & **10** from methylene linker series and compounds **12** & **13** from *N*-aryl series are front runners from this class with excellent ΔT_m_ and MPO profiles.

### 3.3 *In vitro* neuroprotection and toxicity of lead compounds in mouse neuronal cells

**Fig. 3:**
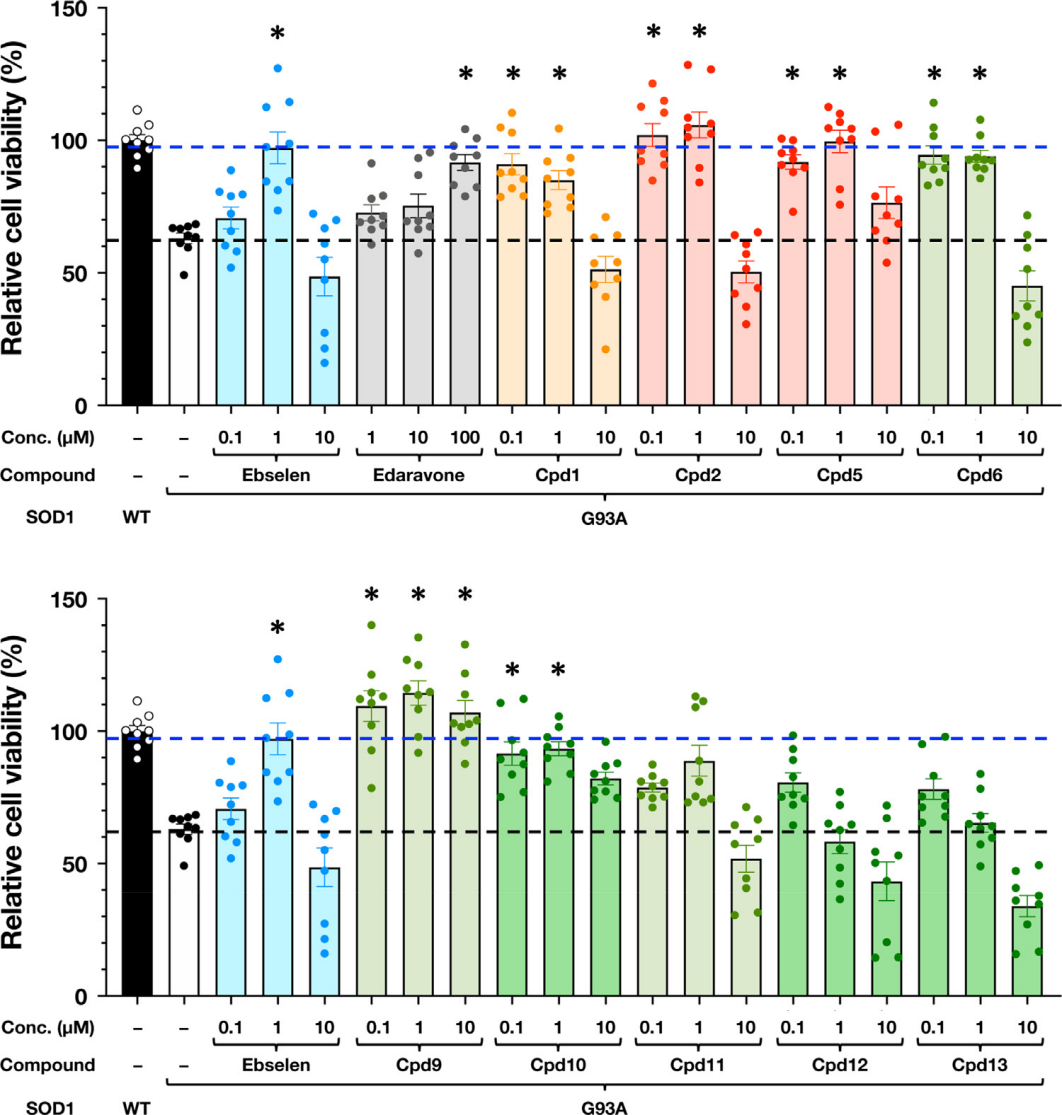
Ebselen-based compounds confer neuroprotection in mouse N2a cells expressing G93A mutant form of SOD1. MTS assays of mouse N2a cells transfected with wild-type (WT) or G93A SOD1 in the presence of ebselen, ebselen-based compounds, or edaravone at the indicated concentrations. The results with each the ebselen-based compounds are displayed with colors based on its physicochemical parameters shown in Table 1. Green – good/desirable; Light green – almost good/desirable; Amber – ok; Red – poor/undesirable. Relative cell viability levels of WT and G93A SOD1 are shown as blue and black dashed lines, respectively. Asterisks indicate statistically significant protection (*p* < 0.05) against G93A SOD1. Averaged cell viability ±SEM relative to the one expressing WT SOD1 was plotted. (For interpretation of the references to colour in this figure legend, the reader is referred to the web version of this article.)

A phenotypic screening of ebselen, edaravone and nine lead compounds (**1, 2, 5, 6, 9, 10, 11-13)** has been undertaken in mouse N2a neuronal cells transfected with human wild-type and G93A SOD1, which is the mutant used in representative SOD1-ALS transgenic mice [27]. The biophysical study of G93A SOD1 revealed that the mutation at residue 93 destabilises SOD1 *via* opening β-barrel instead of impairing dimer interface like A4V [38]. Native mass spectrometry demonstrated that G93A SOD1 has wild-type-like dimer interaction. Ebselen increased dimer affinity of wild-type by only 2 fold and G93A SOD1 by just over 5 fold in contrast to the 60 fold increase for A4V [17]. G93A SOD1 was not used as a model to find dimer stabilising compound *via* DSF assay. However, *in vitro* screen against neuronal cells expressing G93A SOD1 is still critical for verifying neuroprotective activity of compound prior to *in vivo* experiment in SOD1-ALS transgenic mice. All compounds were dosed to N2a cells at the concentrations of 0.1, 1 and 10 μM and then incubated for two days before measuring cell viability by MTS assay. Relative cell viability of each compound at different concentrations are presented in bar charts in Fig. 3. We used N2a cells expressing wild-type SOD1 as a control, because wild-type SOD1 does not induce any obvious damage to cells in this experimental condition [35]. We found that transgenic N2a cells expressing G93A SOD1 used as negative control have significantly lower cell viability, about 62% of wild-type SOD1. Furthermore, edaravone which is an approved ALS drug, was used as positive control in this screen. With the aim of producing an ALS drug that can stabilise SOD1, a good compound should redeem G93A SOD1 transfected cell using a lower dose than edaravone.

Most of the ebselen-based compounds seem to be toxic to N2a cell at 10 μM resulting in lower viability than the negative control. Fortunately, many compounds performed good neuroprotective effect at 0.1 and 1 μM. Compounds **9** and **10** appeared to be the most potent compounds recovering G93A SOD1-transfected cell to the same level of wild-type cell even at 10μM (indicating better overall safety index) and could be selected for *in vivo* experiments. In contrast, compounds **12** and **13,** which possess excellent ΔT_m_ values, gave poor neuroprotection at 1 and 10 μM.

Incorporation of the carbonyl morpholine group in compounds **12** and **13** might not only enhance stabilisation of SOD1 dimer, but also stimulate unexpected cytotoxicity. To verify this concept, cell viability of N2a cells in ebselen, compounds **6** and **12** at seven concentrations were measured and median lethal doses (LC_50_) were calculated. The results in Fig. S5a show that LC_50_ of compound **12** (17.6 μM) was about half of ebselen (32.8 μM), indicating that compound **12** is more cytotoxic than ebselen. On the other hand, compound **6** had LC_50_ at 71.0 μM that is considered as a safer compound than ebselen. To gain further insight, we assayed compound **6** at 0.1 μM in N2a cells expressing other SOD1 mutants- A4V, G37R, G85R (Fig. S5b). This compound was found to be effective not only in G93A SOD1-transfected cell, but also enhanced cell survival in other ALS SOD1 mutants, including A4V. This result suggests that ebselen-based compounds are also applicable and beneficial for neuronal cells expressing other ALS-linked SOD1 mutants.

### 3.4 *In-vivo* experiments: impact of Ebselen in G93A SOD1 transgenic mice

**Fig. 4:**
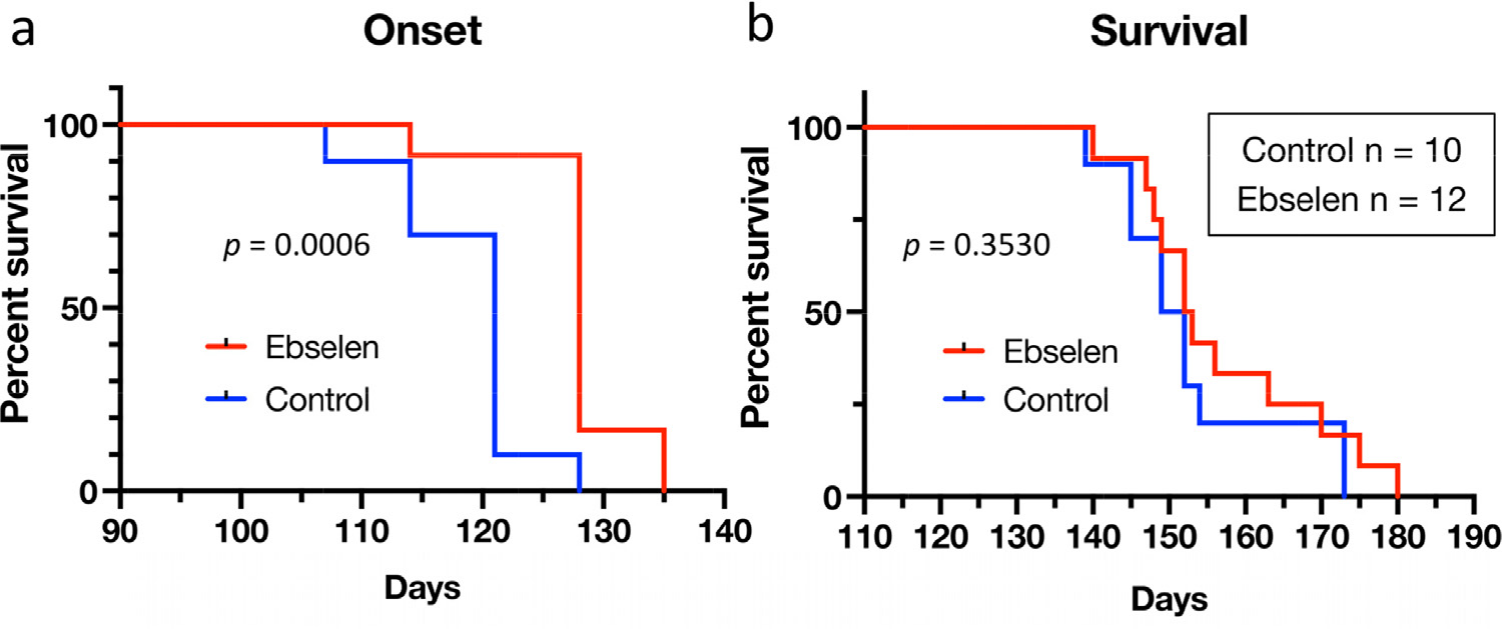
Onset and survival curves of G93A SOD1 transgenic mice treated with ebselen. **a** Disease onset curves for ebselen-treated G93A SOD1 mice and control G93A SOD1 mice (Ebselen: 128.0±5.2 days, Control: 118.9±5.8 days; mean±SD, *p* = 0.0006 in log-rank test). **b** Survival curves for ebselen-treated G93A SOD1 mice and control (Ebselen: 156.6±11.4 days, Control: 153.1±11.4 days; shown as mean±SD, *p* = 0.3530 in log-rank test). The same numbers of mice were used for onset and survival analysis (control, n=10; ebselen, n=12). All the mice are female.

In preparation for selecting potential candidates to progress into the clinical phase, we have set up *in vivo* trial of compounds using G93A SOD1 transgenic mice. The first set of experiments have been completed using ebselen to give an indication of the potential *in-vivo* efficacy of this scaffold and identify potential lead compounds. The mice were fed by powder food mixed with/without 0.016% w/w ebselen (estimated dose: 24 mg/kg) from day 70 till end-stage. Survival experiments were performed to determine disease onset and mouse survival time, shown in Fig. 4. We found that ebselen significantly delayed the disease onset of ALS in mice (control: 118.9±5.8 days and ebselen: 128.0±5.2 days shown as mean±SD). However, mean survival time of transgenic mice was extended only marginally (control: 153.1±11.4 days and ebselen: 156.6±11.4 days, mean±SD). The body weights, the rotarod performance, and the clasping signs, which are the markers used to evaluate disease progression, were not affected by ebselen (Fig. S6).

## 4 Discussion

SOD1 is a ubiquitously expressed protein enriched in nervous system. Genetic mutations throughout the SOD1 structure, including A4V substitution, result in protein destabilisation and cytosolic aggregation leading to neuron death [5]. A4V mutation causes steric clashes with neighbouring residues, altering dimer interface and disturbing protein dimer stability and affinity [9,39,40]. Thus, preventing monomerisation of SOD1 is considered an important feature of novel therapeutic agent that delays SOD1-related ALS progression. Cys111 in SOD1 is amenable to be oxidised that is considered to promote monomerisation and inclusion formation [18,41]. As such it has become a focus for small molecule intervention for stabilising SOD1 dimer and also reducing the chance of oxidative modification [15], [16], [17].

Ebselen has been proven as a potential template with good ability to increase the affinity of A4V SOD1 dimer formation [17] and thermal stability of A4V^C6S^ SOD1 [26] when bound with cys111. In this study, we generated a group of organoselenium compounds using benzoisoselenazolone ring of ebselen incorporated with functionalised methylene linker and *N*-aryl moieties. The new molecules were designed as CNS-accessible compounds with drug-likeness profiles on the basis of high scores of MPO from predicted physiochemical properties (Tables 1&2) before evaluating their performance against A4V^C6S^ SOD1 in DSF assay. Some compounds in methylene linker series (compounds **1, 3, 6, 9, 10** and **14**) and *N*-aryl series (compounds **12** &**13**), which exhibited SOD1 stabilisation with large positive ΔT_m_ values at the same degree or better than ebselen, were listed as potent candidates. Crystallographic investigation of A4V SOD1 bound with those candidates revealed binding poses at cys111 and rationalised how each chemical moiety interacts with SOD1 enhancing dimeric contact. Identical to the parent drug ebselen, *N*-aryl compound **13** localises parallel to loop VI allowing π-π stacking interaction opposing the same molecule at another monomer. The carbonyl morpholine group and fluorine atom in compound **13** can establish water bridging with SOD1 residues at dimer interface through hydrogen bonds that results in fantastic thermal stability. In contrast to *N*-aryl series, the compounds with methylene linker have binding mode almost perpendicular to *N*-aryl compounds due to their higher flexibility. They reinforce dimer stability by picking up interactions with Thr2 and Ile151 of *N*- and *C*-termini of SOD1, respectively. This point could be utilised by growing small functional groups at benzyl tail to develop additional contacts. Incorporation of *para*-methoxy group in compound **3** seems to be more beneficial than halogen atoms according to significant greater ΔT_m_ because its size and position may perfectly fitt the space between Thr2 and Ile151. Nevertheless, *meta*-methoxy group results in distinct poses of benzyl tail in A4V SOD1 dimer. Interestingly, crystal structures of compounds **6** and **9** shows that benzyl tail of one ligand establish cross-monomer linking with Asp109 of opposite monomer. This seems to result in better stabilisation especially in compound **8** promotes thermal stability above other compounds in the same series. Moreover, chemical modification using a pyridyl ring as a more polar aromatic tail in compound **9** confers exceptional values of MPO scores and physicochemical parameters that opens opportunity to explore more potential compounds based on this moiety. We modified compound **9** by incorporating with a *para*-methoxy group (compound **10**) or carbonyl morpholine (compound **14**). Both compounds certainly gave the highest rank in MPO scores and other parameters and effective A4V SOD1 stabilisation which may be potential neuroprotective agents with expected good drug-likeness.

We tested the neuroprotective activity against G93A SOD1 expressing mouse neuronal cells of potent candidates which were selected by exceptional ΔT_m_, MPO and physiochemical parameters scores. The parent ebselen can redeem G93A SOD1 neuron cell viability almost up to wild-type level in much lower dose than the licensed drug edaravone, which implies better potency for this class of compounds. We can see potentially high survival level of neuronal cells after treatment with low concentrations at 0.1 and 1 μM of all methylene linker compounds. However, most compounds in this series and ebselen show lethality to neuron cells at concentration of 10 μM. Compounds **9** and **10** containing a pyridyl ring seems to be the most promising candidates among tested compounds with the strongest *in vitro* neuroprotective activity and much smaller loss of neuron cells at high dose. Thus, the methylene linker compounds with pyridyl group should be the focus for further development and optimisation of safe neuroprotective compounds, but *para*-methoxy group appears not essential to improve healing effect in mouse neuronal cells. Carbonyl morpholine derivatives (compounds **12, 13**), which are the most promising candidates due to their excellent ΔT_m_ and predicted MPO scores, exhibit the poorest level of neuroprotection among other candidates. This observation suggests carbonyl morpholine group displays non-selective binding to other endogenous targets leading to neuron cell death.

In addition to *in vitro* neuroprotective performance of our compounds, transgenic mice expressing G93A SOD1, a frequently used SOD1-ALS mouse model, were treated orally with ebselen and monitored for their motor activities and survival times. Even though ebselen does not extend total lifetime of experimental mice appreciably compared to the controls, we observed a delayed disease onset in ebselen-treated G93A mice (>10 days). This confirms that ebselen can be absorbed in digestive tract and replicate its neuroprotective effect in animal model. Ebselen has a reducing activity to suppress oxidative stress that may contribute to neuroprotective effect [25]. However, our compounds, apart from their reducing potential, showed significantly improved neuroprotective activity measured by our MTS assay. This suggests that the direct interaction with SOD1 is a key determinant of neuroprotective activity of our new compounds rather than their reducing activity per se. We propose that compounds **9** and **10** may prove better in the *in vivo* activities than ebselen and result in an increased survival rate of transgenic mice and thus may have better prospects for clinical trials.

Thus, we have generated two promising series of ebselen-based compounds for stabilising mutant SOD1 including A4V. We used DSF assays, MPO scores and predicted physicochemical parameters to rank lead compounds before measuring neuroprotective effects in cell-based assays and animal models. The methylene linked series showed outstanding neuroprotective activities in mouse neuronal cells, particularly compounds **9** and **10** with a pyridyl tail, which can be considered an excellent scaffold for further optimisation in drug development programme. However, our *N*-aryl compounds with carbonyl morpholine are less selective in spite of excellent outcomes in DSF assays. These results demonstrate that DSF assay is a good tool for hit to lead optimisation in this disease area, but phenotypic cell-based experiments are still required to evaluate *in vitro* effectiveness and cytotoxic effects. Apart from neuroprotection and safety profiles, *in vitro* and *in vivo* pharmacokinetic and pharmacodynamics studies of lead compounds should be addressed to select the most powerful candidates. Here, we also verified neuroprotective action of ebselen in mouse model showing effective oral availability and delay in disease onset. Further optimisation of this template can be envisaged to improve drug potency and increase the overall survival time.

## 5 Conclusions

We have established that the new generation of ebselen-based compounds are potent, multimodal SOD1 pharmacological chaperone targeting cysteine 111, restoring the dimer affinity of, say A4V SOD1 to that of uncompromised wild-type SOD1, prevent the formation of oligomers/aggregates. This is an important significant milestone in the chemical-assisted manipulation of SOD1 for the treatment of ALS endorsed by the neuroprotection offered in mouse N2a neuronal cells transfected with human wild-type and G93A SOD1. Several of the compounds including ebselen redeemed G93A SOD1 transfected cells better than the drug Edaravone (approved in USA in 2017), which has had limited efficacy and acceptance as a drug worldwide, requiring daily intravenous infusion. In addition to the *in vitro* neuroprotective performance, oral treatment with ebselen did not extend total lifespan of G93A SOD1 mice, but significantly delayed a disease onset by >10 days, confirming that ebselen (and by extension ebselen-based compounds) can be absorbed in digestive tract and replicate its neuroprotective effect in animal model. In summary, we have demonstrated outperformance of ebselen and its new generation of leads over edaravone where the formulations are suitable for effective oral administration for a sustained treatment for ALS.

The ability of our compounds to target a cysteine may extend to other protein targets such as the main protease (M^pro^) of COVID-19 virus whose recent crystallographic studies with inhibitor and the docking study with ebselen indicates covalent interaction with Cys145 is the mechanism of its inhibition [42]. This is further supported by the docking of new ebselen-based compound developed here, Fig. S7.

## Author contributions

Conceptualization, S.S.H. and P.M.O.; Methodology, K.A., M.C.R., and S.W.; Investigation, K.A., M.C.R., S.W., S.S.H., P.M.O. and K. Y.; Writing – Original Draft, K.A., M.C.R. and S.W.; Writing – Review & Editing, S.S.H., P.M.O. and K. Y.; Funding Acquisition, S.S.H., P.M.O. and K. Y.; Supervision, S.S.H., P.M.O. and K. Y. All authors read and approved the final version of the manuscript.

## Declaration of Interests

The authors declare no competing interests.

## Appendix Supplementary materials

Image, application 1
