# Supplementary material for: Novel Selenium-based compounds with therapeutic potential for SOD1-linked amyotrophic lateral sclerosis
Source: eBioMedicine. 2020 Aug 30;59:102980. doi: 10.1016/j.ebiom.2020.102980 (PMC7456458; doi:10.1016/j.ebiom.2020.102980)
Supplement: Supplementary file 1 [file mmc1.docx]

**Supplementary Information**


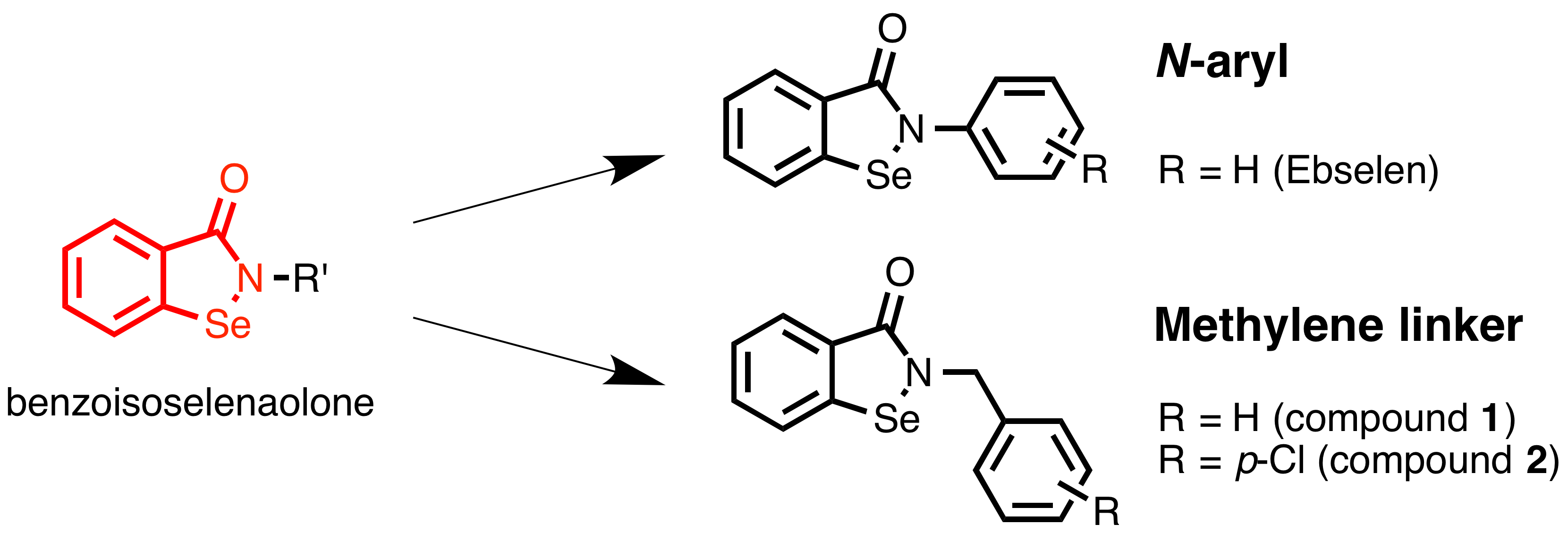


Fig S1. Benzoisoselenazolone core (red) split into two scaffolds: methylene linker and the other *N*-aryl. Ebselen is based on the *N*-aryl scaffold with R= Ph.


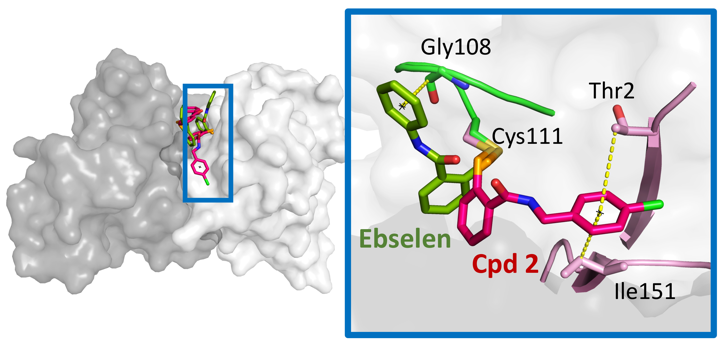


Fig S2. Binding modes of ebselen and compound **2** at dimer interface of A4V SOD1 visualised by crystal structures in C2 space group (PDB: 6SPH and 6SPI, respectively). SOD1 monomers are displayed as light and dark grey surface. Binding site at cys111 is highlighted in blue box. Molecules of ebselen and compound **2** are illustrated as green and deep pink sticks. Loop VI (residues 106-111) is shown as light green cartoon and sticks, and N- and C- termini are shown as light pink cartoon and sticks. Close contacts (<4.5Å) between protein and ligand are highlighted as yellow dashing lines.


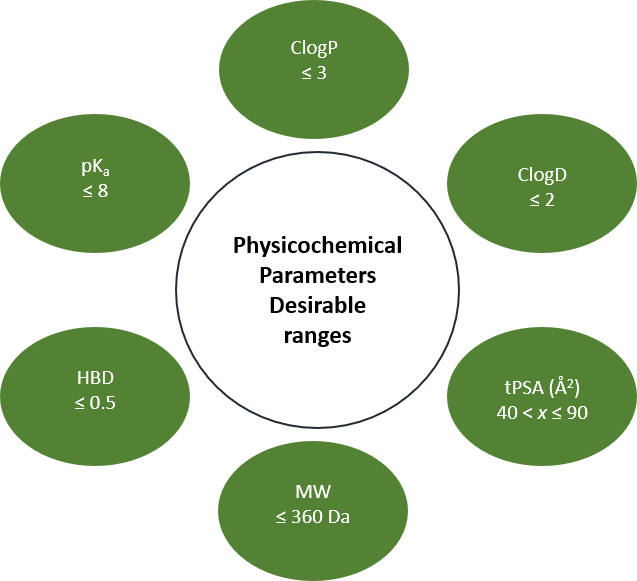


Fig S3. Desirable ranges of physiochemical parameters for good drug-like compound. ClogP - calculated partition coefficient, ClogD - calculated distribution coefficient, MW - molecular weight, tPSA - topological polar surface area, HBD - hydrogen bond donor, pKa - acid dissociation constant**.**


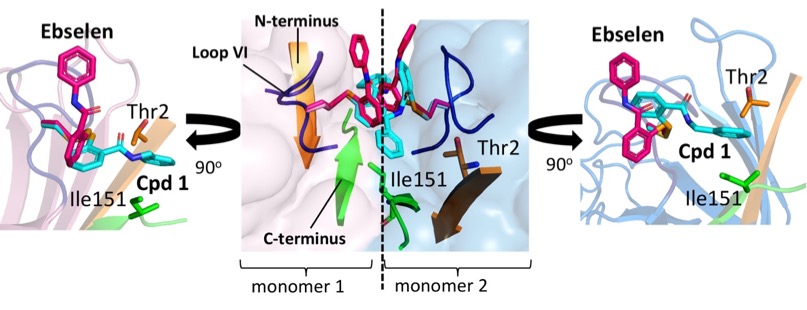


Fig S4. Superimposed crystal structures of SOD1 dimer bound with ebselen (pink sticks) and compound **1** (cyan sticks) in demonstrating different binding poses within the dimer interface groove.


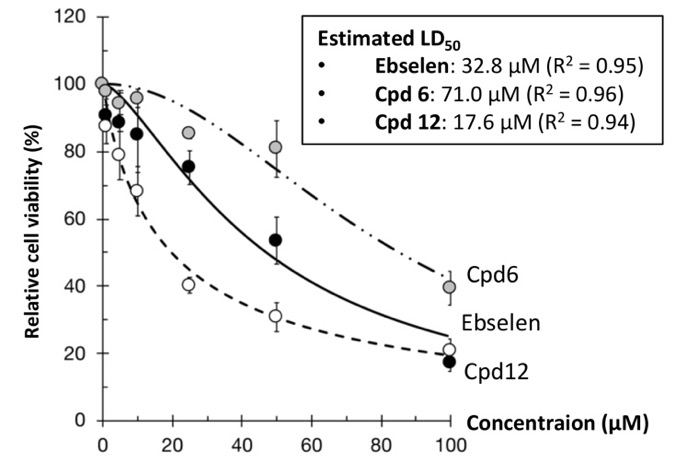


**a**


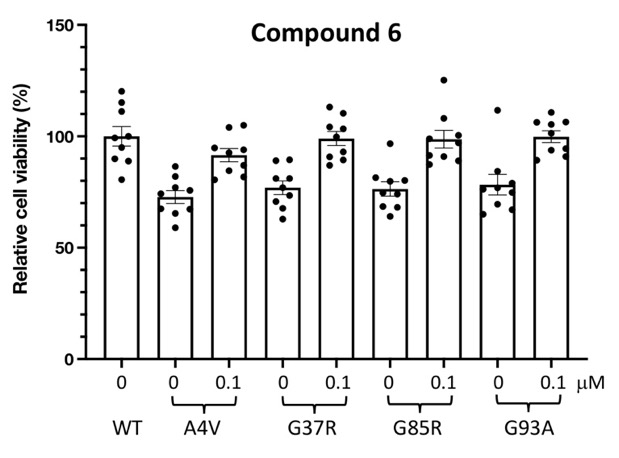


**b**

Fig S5. **a** LC_50_ determination of ebselen, compounds **6** and **12** in N2a cells. The N2a cells were incubated for 48 h in the presence of the compounds at the indicated concentrations. Relative cell viabilities were determined by MTS assay and normalized by the viabilities at 0 µM controls. Data are shown as mean ± SEM (n = 3, triplicate). **b** MTS assays of N2a cells transfected with wild-type (WT), A4V, G37R, G85R and G93A SOD1 treated with 0.1μM compound **6**. Mean cell viability ± SEM relative to the one expressing WT SOD1 was plotted (B).


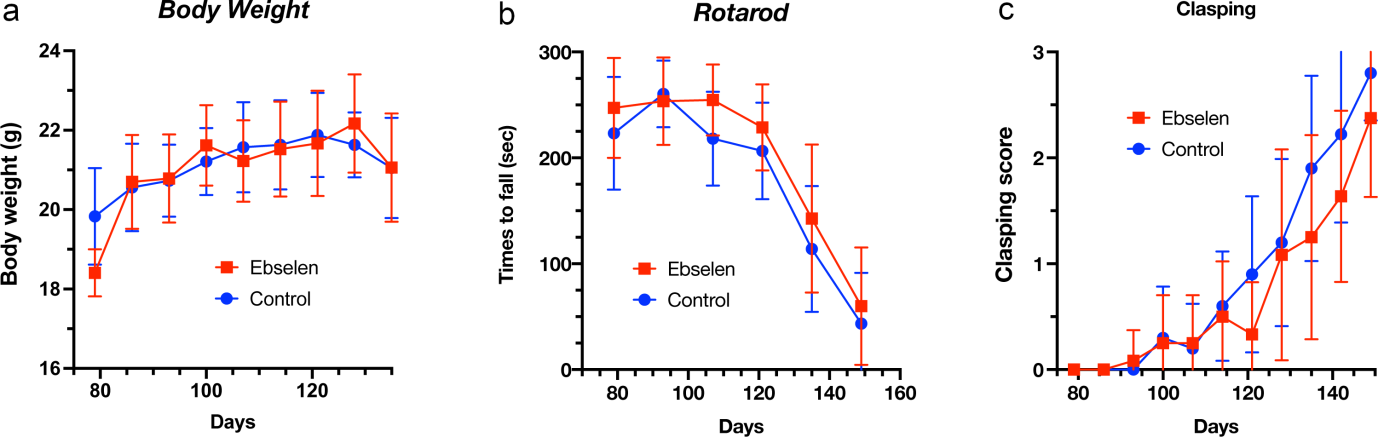


Fig S6. Body weights, motor performance, and clasping phenotype were not significantly altered in G93A SOD1 mice with ebselen treatment.

**a** Mean body weight, **b** rotarod performance, and **c** clasping scores were plotted for G93A SOD1 mice treated with or without ebselen. Data are shown as mean ± SD. n = 10 for the control and 12 for the ebselen-treated group. All the mice are female.


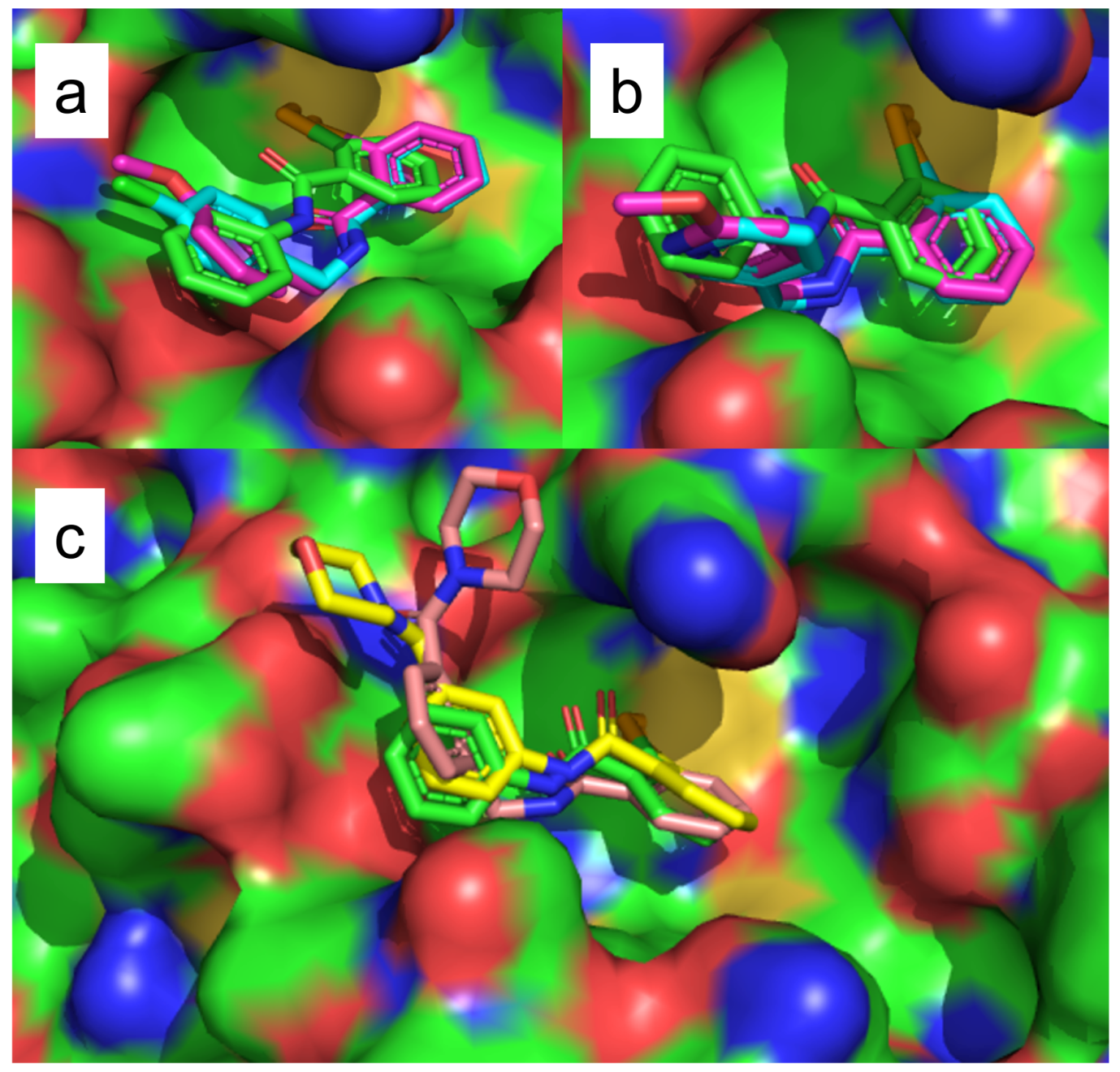


Figure S7. Overlay of docking images each with Ebselen. (A) Overlay of compounds **2** & **6** with the methylene linker which matches ebselen docking. (B) Overlay of compounds **9** & **10** with the methylene linker which shows a different orientation for the pyridyl ring compared to the phenyl of ebselen which could be capable of providing an additional binding interaction and could rationalise the increased PLP fitting score matches ebselen docking. (C) Overlay of compounds **11** & **14** which contain the carbonyl morpholine moiety which occupies more of the binding site where further interactions could be obtained and rationalise the increased PLP fitness score (>75) compared to fitness score of 66 for ebselen. The surface of the protein was coloured as followed Carbon: Green, Nitrogen: Blue and Oxygen: Red. X-ray crystal structure (PDB: 6YB7) was rendered in PyMOL Molecular Graphics System, Version 2.2.2, Schrödinger, LLC.

Table S1. Primer sequences used for generating A4V and A4V C6S human SOD1 mutant genes in pET303C plasmid.

| **Mutant** | **Forward Primer Sequence (5’ to 3’)** | **Reverse Primer Sequence (5’ to 3’)** |
| --- | --- | --- |
| A4V | ATGGCGACGAAGGTCGTGTGCGTGCTG | CAGCACGCACACGACCTTCGTCGCCAT |
| A4V C6S | ATGGCGACGAAGGTCGTGAGTGTGCTG | CAGCACACTCACGACCTTCGTCGCCAT |

**Supplementary methods**

***Analyses of the phenotype and motor performance of G93A SOD1 mouse***

The body weight of the mice and clasping signs were measured every week. The clasping signs were scored from 0 (no sign) to 3 (most severe), as described elsewhere (Guyenet et al. 2010). Rotarod tests were performed every two weeks, as previously described (Watanabe et al. 2016). Briefly, the mice were challenged to 0.01 rpm/min acceleration for 5 min. The best time in three trials with each 15 min interval was scored. All the results are analysed by two-way ANOVA following multiple comparison tests using Prism 8 software.

***Docking study of compounds with M^pro^-COVID19***

The small molecule 3D structures were built using Spartan '14 v1.1.8 (http://computationalchemistry.co.uk/spartan10.html) and energy minimised using MMFF forcefield. Molecular docking of the small molecules into the active site of M^Pro^ was performed using GOLD Suite v5.21. The crystal structure of M^Pro^ was downloaded from the PDB (ID: 6YB7) and was prepared for use in docking calculations by addition of hydrogen atoms in the protein and crystallographic water molecules located in the active site. The active site for docking was defined to be selecting sulphur atom of the Cysteine 145 residue for covalent docking. Each ligand was set to undergo 10 GA runs with no early termination allowed, lone pairs were not saved and all solutions were kept. The rest of the settings were left as default. Goldscore and ChemPLP fitness function were the output obtained.

**Supplementary references**

Watanabe, S. et al. (2016). Mitochondria‐associated membrane collapse is a common pathomechanism in SIGMAR 1 ‐ and SOD 1 ‐linked ALS. EMBO Mol. Med. 8, 1421–1437.

Guyenet et al. (2010) A Simple Composite Phenotype Scoring System for Evaluating Mouse Models of Cerebellar Ataxia J. Vis. Exp. 21(39), 1787.
